# The influence of *Helicobacter pylori*, proton pump inhibitor, and obesity on the gastric microbiome in relation to gastric cancer development

**DOI:** 10.1016/j.csbj.2023.11.053

**Published:** 2023-11-30

**Authors:** Chengliang Zhou, Tanya M. Bisseling, Rachel S. van der Post, Annemarie Boleij

**Affiliations:** a Radboud University Medical Center, Radboud Institute for Molecular Life Sciences, Department of Pathology, P.O. box 9101, 6500 HB Nijmegen, the Netherlands; b Radboud University Medical Center, Radboud Institute for Molecular Life Sciences, Department of Gastroenterology and Hepatology, P.O. box 9101, 6500 HB Nijmegen, the Netherlands

**Keywords:** Gastric microbiome, Non-*Helicobacter pylori* bacteria, Obesity, PPI use, 16 s rRNA sequencing

## Abstract

*Helicobacter pylori* infection is still the main risk factor for the development of gastric cancer (GC). We explore the scientific evidence for the role of the gastric microbiome beyond *Helicobacter pylori* (*H. pylori*) in gastric carcinogenesis. The composition of the gastric microbiome in healthy individuals, in presence and absence of *H. pylori* infection, in proton pump inhibitor (PPI)-users, obese individuals, and GC patients was investigated. Possible mechanisms for microbial involvement, limitations of available research and options for future studies are provided.

A common finding amongst studies was increased levels of *Streptococcus*, *Prevotella*, *Neisseria*, and *Actinomyces* in healthy individuals or those with *H. pylori-*negative gastritis*.* In PPI-users the risk for GC increases with the treatment duration, and the gastric microbiome shifts, with the most consistent increase in the genus *Streptococcus*. Similarly, in obese individuals, *Streptococcus* was the most abundant genus, with an increased risk for cardia GC. The genera *Streptococcus, Lactobacillus* and *Prevotella* were found to be more prominent in GC patients in multiple studies. Potential mechanisms of non-*H. pylori* microbiota contributing to GC are linked to lipopolysaccharide production, contribution to inflammatory pathways, and the formation of N-nitroso compounds and reactive oxygen species.

In conclusion, the knowledge of the gastric microbiome in GC is mainly descriptive and based on sequencing of gastric mucosal samples. For a better mechanistic understanding of microbes in GC development, longitudinal cohorts including precancerous lesions, different regions in the stomach, and subtypes of GC, and gastric organoid models for diffuse and intestinal type GC should be employed.

## 1 Introduction

The human gastrointestinal (GI) microbiome constitutes a complex ecosystem and is an integral aspect of human biology [1], [2]. Greater microbial richness and bacterial diversity are associated with better overall health and less comorbidity, such as metabolic diseases and obesity [3], [4]. The gut microbiota colonizes the whole GI tract, including the stomach*.* However, the gastric environment is particularly harsh and difficult to colonize, mainly because of its low pH value. As a result, the gastric microbial load is much lower compared to the small intestine or the colon [5]. Since methodological advancements have given us a more detailed view of the gastric microbiome through culture-independent approaches, the hypothesis that the stomach is a hostile, sterile environment has changed [6], [7]. Compositional changes in gastric mucosal microbiota have been associated with an increased risk of GC, but also with the development of other gastric diseases [8], [9].

**Fig. 1:**
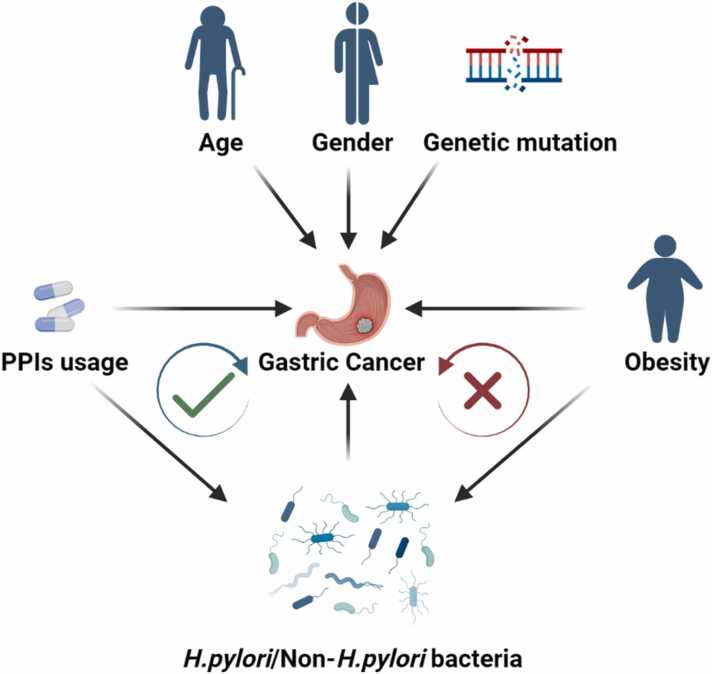
The logical relationship between obesity and PPI use as factors influencing the development of GC and other impact factors (such as age, gender, genetic mutation).The green circles indicate that PPI does affect the development of GC by altering the microbial composition, while the red circles indicate that only studies have shown that obesity does affect the microbial composition of the stomach and also affects the development of GC, but whether the altered microbes are involved in the cancerous process remains to be explored.

GC is a global health problem with a poor prognosis and high mortality; it represents the fifth most common malignancy in the world with 769.000 deaths, the third leading cause of cancer-related mortality[10]. Changes in the composition of the gastric mucosa, especially infection with *H. pylori* may lead to the development of gastric abnormalities [11], [12]. GC is a multifactorial disease, where many factors can influence its development, including environmental factors, age, gender, dietary habits (high-fat diet, salt), medication (*H. pylori* eradication, proton-pump inhibitor (PPI)), bacterial infection (*H. pylori* and non- *H. pylori* bacteria), low socio-economic status, geographical differences and inherited gene alterations. GC development and progression is a multiannual and multistage process [13]. The incidence of GC is higher in males than in females before menopause with a male-to-female ratio of between 2:1 and 3:1, but after menopause, the incidence is similar between males and females [14], [15]. The incidence of GC in the elderly is high, but according to the latest epidemiological statistics, the incidence of GC among young adults (aged <50 years) in both high and low-risk countries is increasing compared to the elderly [15]. The highest GC incidence rates are in Eastern Asia (Mongolia, China, South Korea, and Japan), the Andean regions of South America, and Eastern Europe. The lowest incidence rates are in North America, Northern Europe, and most countries in Africa and South-eastern Asia. A large amount of evidence supports the connection between GC and risk factors: *Helicobacter pylori* and the gastric micro-environment hold a significant position in the development of GC (Fig. 1).

Among GC risk factors, we have found that bacterial infection, dietary habits as well as medication history (*H. pylori* eradication) can greatly alter the state of *H. pylori* and the microenvironment in the stomach. *H. pylori,* belonging to the γ-*Proteobacteria*, is a major risk factor for the development of GC and has been associated with the precancerous steps of chronic gastritis, atrophy, intestinal metaplasia and finally the intestinal type of GC [16]. However, gastric microbes other than *H. pylori* might also participate in gastric diseases and carcinogenesis [17], [18], [19]. While *H. pylori* significantly contributes to the risk of GC, *H. pylori* eradication doesn't fully remove the potential for GC development. Studies indicate that eradication of *H. pylori* decreases the risk of GC by 33%− 47% [20]. PPI usage plays an important role in *H. pylori* eradication therapy and increases bacterial abundance. We therefore explored the relationship between eradication therapy, PPI use, and the mucosal microbiome in relation to GC (Fig. 1). Similarly, there is a strong link between obesity and GC. One of the risk factors for obesity includes a high fat and sodium diet, which can result in microbial dysbiosis and promote the development of various malignancies [21]. Because of the clear link between obesity and the risk for GC and obesity results in fecal microbial dysbiosis, we envisioned that obese individuals might also have a changed gastric mucosal microbiome potentially affecting the development of GC (Fig. 1). To conclude, *H. pylori*, obesity and use of PPIs have been linked to an increased risk of developing GC and may change the gastric mucosal microbiota.

The gastric microbiome has been investigated in high-quality studies with different sampling sources including gastric juice, mucosal brush samples, gastric biopsies, and even fecal samples. Both fecal samples and gastric juice may not appropriately reflect the gastric mucosal microbiome in relation to GC. GC originates from mutations in stem cells from mucosal epithelial origin, therefore, in this review we focus on all studies considering gastric microbiota from mucosal biopsies. The mucosal microbiome in GC patients differs from healthy individuals [17], [19], these bacteria may synthesize toxic metabolites and potentially induce oxidative and nitrosative DNA damage. Nitrosative compounds and oxidative stress can cause atrophy, chronic inflammation, cell proliferation, and subsequently dysplasia, resulting in GC [22], [23]. Nevertheless, little is known about the impact of non-*H. pylori* microbiota on GC development.

In this review we give an overview of the gastric microbiome in the presence and absence of *H. pylori* and the changes that occur in the gastric microbiome of patients with obesity, and PPI usage, and how this may lead to GC. Furthermore, we will describe possible mechanisms for microbial involvement in GC development, describe limitations of the current available research and provide options for future studies to get a better understanding of microbes in GC development.

## 2 Search strategy

In the PubMed database a search was performed for articles that investigated the composition of the gastric microbiome in healthy individuals, PPI users, individuals with obesity and GC patients. Studies that analyzed the bacterial diversity within the gastric mucosa by using 16 S rRNA analysis or metagenome analysis were included. We compared the normal gastric microbiome with the microbiota in PPI users, obesity, and GC, both in presence and absence of *H. pylori*. We involved articles published between January 2000 until April 2023. Search MESH terms were stomach microbiome, *Helicobacter pylori*, non-*H. pylori*, gastric cancer, gastritis, proton pump inhibitor, obesity, BMI, mucosa, and biopsy. Only articles published in English were included. The PRISMA flow diagram of the selected studies for each search query is summarized in Supplemental figure.

## 3 Risk factors for gastric cancer & microbiome

### 3.1 Pathogenic *Helicobacter pylori* infection

*H. pylori is* the first bacterium discovered to inhabit the gastric mucosa that can survive in the acid environment of the stomach and is the most frequent cause of chronic gastritis [24]. Since Marshall and Warren isolated *H. pylori* from the gastric mucosa in the early eighties, the stomach was no longer considered a sterile environment [8]. *H.* pylori, a spiral-shaped, microaerophilic, gram-negative bacterium, can colonize the human stomach by moving through the gastric mucus layer, and may establish long-term colonization [25]. *H. pylori* has a variety of virulence factors, including cytotoxin associated gene A (CagA) and vacuolating cytotoxin A (VacA). The CagA protein, transported from *H. pylori* into host cells through the Cag IV secretion system (T4SS), perturbs multiple downstream signaling pathways, resulting in cytopathic effects and subsequent cell transformation. VacA constitutes one of the most crucial virulence factors that enable bacterial colonization and survival in the gastric epithelium; the VacA gene is present in all the *H. pylori* strains. The VacA-s1m1 genotype is commonly found in *H. pylori*-infected patients with chronic gastritis, whereas in *H. pylori*-induced GC, usually vacA-s1 and vacA-m1 allelic variants strongly increased susceptibility to GC [26], [27]. The release of virulence factors following *H. pylori* infection of gastric epithelial cells can activate nuclear factor κB (NF-κB), extracellular signal-regulated kinase/mitogen-activated protein kinase (ERK/MAPK), cytokine-stimulated transduction (JAK-STAT) signaling pathway, and be involved in processes that impair gastric epithelial cells such as methylation, apoptosis and necrosis, which can control the expression of a number of host proteins and influence the growth of gastric epithelial cells [28].

Chronic *H. pylori* infection causes mucosal inflammation and induces histological changes and is considered a major risk factor for GC. Nevertheless, only 3% of people with *H. pylori* infections will progress to GC, suggesting the importance of other factors in gastric carcinogenesis [11]. In Correa’s model of gastric carcinogenesis, infection with *H. pylori* induces chronic active gastritis, and may lead to atrophic gastritis, intestinal metaplasia, gastric precancerous lesions (i.e. low- and high-grade dysplasia) and finally progression to GC and/or gastric mucosa-associated lymphoid tissue (MALT) lymphomas [16]. *H. pylori* penetrates the mucus layer by its motility and survives in a niche between the gastric epithelial cells and the mucus [12]. In antral-predominant gastritis, acid secretion usually remains intact and *H. pylori* colonization is limited to the antrum. In corpus-predominant gastritis there is low or absent acid secretion which may lead to severe atrophic gastritis, intestinal metaplasia, and GC. *H. pylori* infection typically starts in the distal stomach (i.e. antropyloric region) and without treatment may move upwards to the gastric corpus*. H. pylori* produces ammonia and bicarbonate from urea, elevating the gastric pH, which may allow growth of other bacteria [29], [30], [31], [32], [33]. Furthermore, *H. pylori* can reduce gastric motility, reducing clearance of toxins or bacteria from the gastric mucosa [34]. The interaction of *H. pylori* with other gastric microbiota may also play a role in gastric carcinogenesis [29]. These facts could all potentially lead to the growth of other non-*H. pylori* bacteria. In the next paragraph, we will discuss the current knowledge about the human gastric microbiota beyond *H. pylori*.

### 3.2 Non-*H. pylori* microbiota in the stomach

Culture-independent technological advancements have given us a first view of the composition of the gastric microbiota in the absence and presence of *H. pylori* [25]. An overview of the studies that have assessed the composition of the human gastric microbiome is given in Table 1. We included 11 articles that used culture-independent methods to identify individual gastric microbiomes of healthy participants without gastritis and patients with gastritis that were subdivided in *H. pylori* positive and negative individuals. The specifics for each study are given in Table 1. Eight studies considered the human gastric microbiome in *H. pylori* positive and negative individuals. For the subject of this study, we extracted information from each article on gastric microbiome composition in three conditions, which were healthy individuals, and gastritis patients that were either *H. pylori* positive or negative.

**Table 1:** The composition of non-*H. pylori* gastric microbiota in stomach in absence and presence of *H. pylori*.

Study	*H. pylori* + /- individuals	Dominant gastric microbiota		Patient characteristic	Detection method/Platform
Wang *et al.* (2022)[38]	*H. pylori* (-) *H. pylori* (+)	Phylum level	Species level	Total patients (N = 96) 12 of 96 with chronic active gastritis, 54 of 96 with chronic gastritis, 30 of 96 unclassified	Metagenomic sequencing /DNBSEQ‐T1 platform (MGI, Shenzhen, CHN)
		*H. pylori* (-): *Proteobacteria* (77.98%) *Bacteroidetes* (13.35%) *Actinobacteria* (3.94%) *Firmicutes* (4.55%) *H. pylori* (+): Proteobacteria (88.43%) Bacteroidetes (5.59%) Actinobacteria (3.75%) Firmicutes (2.15%)	*H. pylori* (-): *Stenotrophomonas maltophilia*, *Pseudomonas unclassified* *H. pylori* (+): *Helicobacter pylori*		
Yuan *et al.* (2021)[45]	*H. pylori* (-) (N = 56) *H. pylori* (+) (N = 95)	*H. pylori* (-): The most prevalent genera were *Streptococcus, Helicobacter, Neisseria, Alloprevotella, Prevotella,* and *Actinomyces*, *Appendix* *H. pylori* (+): The most prevalent genera were *Helicobacter*, *Bacteroides*, *Faecalibacterium*, *Blautia*,*Prevotella*, and *Bifidobacterium*		Total patients diagnosed as normal or chronic superficial gastritis	16 s rRNA sequencing /Illumina Novaseq6000 platform
Mao *et al.* (2021)[40]	*H. pylori* (-) (N = 23) *H. pylori* (+) (N = 17)	Phylum level	Genus level	Total patients diagnosed as chronic gastritis	16 s rDNA sequencing /NovaSeq PE250
		*H. pylori* (-): *Firmicutes, Proteobacteria,* *Actinobacteria, Bacteroidetes, Fusobacteria* *H. pylori* (+): *Epsilonbacteraeota, Proteobacteria, Firmicutes, Bacteroidetes, Actinobacteria*	*H. pylori* (+): *Campylobacteria (class), Campylobacterales (order), Helicobacteraceae (family), Helicobacter(genus)* *H. pylori* (-): *Streptococcus, Bifidobacterium, Escherichia-Shigella, Collinsella, Ruminococcus gnavus group, Neisseria, Pseudomonas, unclassified Mitochondria* (genus)		
Miftahussurur *et al.* (2020)[39]	*H. pylori* (-) (N = 110) *H. pylori* (+) (N = 22)	Family level	Species level	Total patients diagnosed as chronic gastritis or intestinal metaplasia	16 s rRNA sequencing /next-generation sequencer MiSeq platform
		*H. pylori* (-): *Streptococcaceae Prevotellaceae Veillonellaceae* *H. pylori* (+): *Helicobacteraceae*	*H. pylori* (-): *Prevotella melaninogenica*(23.5%) *Rothia mucilaginosa*(19.5%) *Veillonella dispar*(18.6%) *Haemophilus parainfluenzae*(7.2%) *H. pylori(+): Helicobacter pylori*		
He *et al.* (2019)[41]	*H. pylori* (+) (N = 10) *H. pylori* (-) (N = 7)	Phylum level	Genus level	10 patients diagnosed *H. pylori*-related gastritis; 7 without *H. pylori* infection gastritis as healthy controls.	16 s rDNA sequencing /Library Quantification Kit for Illumina (Kapa Biosciences, Woburn, MA, USA)
		*H. pylori* (+): Increase in the abundance of *Proteobacteria*, *Actinobacteria*, and *Acidobacteria* compared with *H. pylori* (-) group	*H. pylori* (+): Decrease in the abundance of *Subdoligranulum*, *Lachnoclostridium* compared with *H. pylori* (-) group; Increase in the abundance of *Alistipes* compared with *H. pylori*(-) group		
Delgado *et al.* (2013)[5]	*H. pylori* (-) (N = 4)	The most abundant operational taxonomic units belonged to *Lactobacillus*, *Streptococcus*, and *Propionibacterium.*		Total patients diagnosed as dyspepsia, and also determined to be *H. pylori* negative.	Culturing in combination with Pyrosequencing/ABI 373 DNA sequencer
Maldonado-Contreras *et al.* (2011)[42]	Healthy individuals: *H. pylori* (+) (N = 8) *H. pylori* (-) (N = 4)	*H. pylori* (+): dominated by *H. pylori* and *Proteobacteria, Spirochetes*, *Acidobacteria* *H. pylori* (-): *Actinobacteria*, *Firmicutes*		Tolal patients diagnosed as gastritis or dyspepsia	16 S rRNA sequence /G2 PhyloChip
Li *et al.* (2009)[37]	Antral gastritis (N = 5) Healthy individuals (N = 5)	Antral gastritis: *Streptococcus*, *Firmicutes* Healthy individuals: *Streptococcus*, *Prevotella*, *Porphyromonas*, *Neisseria*, *Haemophilius*		Total patients determined to be *H. pylori* negative.	16 S rRNA sequence /ABI 3730xl sequencer (Applied Biosystems)
Andersson *et al.*(2008)[43]	*H. pylori* (+) (N = 3) *H.pylor i*(-) (N = 3)	*H. pylori* (+): dominated by *Proteobacteria* *H. pylori* (-): dominated by *Streptococcus*, *Actinomyces*, *Prevotella*, *Gemella*, *Chlamydia*, *Cyanobacteria* (phylum level)		Total patients were healthy individuals	Pyrosequencing /Genome Sequencer 20 system (Roche, Basel, Switzerland)
Bik *et al.* (2006)[25]	Healthy individuals: *H. pylori* (+) (N = 19) *H. pylori (-)* (N = 4)	Dominated by *Firmicutes*, *Actinobacteria*, *Bacteroidetes*, *Fusobacteria*, and *Proteobacteria*		Total patients were healthy individuals	16 S rRNA sequence /ABI 3730xl sequencer (Applied Biosystems, Foster City, CA)
		No significant difference between *H. pylori* positive and negative individuals in phylum or genera level			
Monstein *et al.* (2000)[46]	H pylori (+) gastritis (N = 8) healthy individuals (N = 5)	All *H. pylori* positive individuals: *Enterococcus*, *Pseudomonas*, *Streptococcus*, *Staphylococcus*, *Stomatococcus*		8 patients diagnosed as gastritis;5 patients as healthy controls.	TTGE, 16 S rDNA sequence /SEQ4×4 Personal Sequencing System (Amersham-Pharmacia Biotech)

**Fig. 2:**
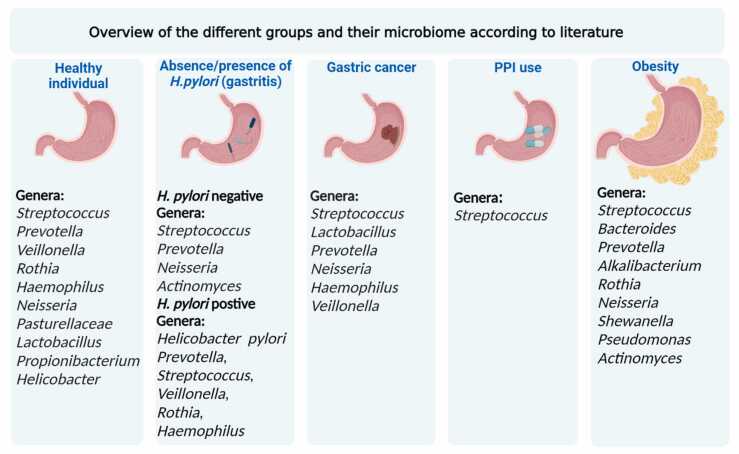
Overview of the different groups and their microbiome according to literature. This figure shows the most dominant bacteria in each category. Healthy individual was described as patients without gastritis. In the absence /presence of *H. pylori* group, the patients were all diagnosed as gastritis and the most dominant bacteria were classified by *H. pylori* status.

In healthy individuals (*H. pylori* negative healthy individuals without gastritis) the gastric microbiome is predominant of the phyla *Proteobacteria*, *Bacteroidetes*, *Firmicutes, Fusobacteria* and *Actinobacteria* [35], but the most prominent phylum can differ between individuals. The most prevalent genus is *Streptococcus* followed by *Prevotella, Veillonella, Rothia,* and *Haemophilus* [36] (Table 1, Fig. 2). *Streptococcus* and *Prevotella* represented 40.6% and 41.5% of all clones respectively in two studies [25], [37].

In 4 studies comparing *H. pylori* positive with negative individuals (these negative individuals were described as gastritis without *H. pylori* infection), an increased microbial richness and higher Shannon diversity was found in *H. pylori* negative individuals [38], [39], [40], [41]*.* Besides a reduced α‐diversity of the gastric community, the overall composition of the gastric microbiota in *H. pylori*‐infected individuals was distinctly different from the negative controls. In contrast, an earlier study by Bik *et al.* was unable to show difference in microbial abundance and α‐diversity in the presence of *H. pylori* [25], however, this might be explained by the small sample size compared to more recent studies. In the *H. pylori* positive group, *H. pylori* dominated the gastric microbiome and presence of *H. pylori* was significantly associated with presence of other *Proteobacteria*, *Acidobacteria* and *Spirochetes* in corpus biopsy samples [42]. The relative abundance of *Proteobacteria*, *Actinobacteria*, and *Acidobacteria* were significantly increased in the *H. pylori* positive group compared to the *H. pylori* negative controls [41]. Wang *et al.* investigated gastric swabs of Chinese patients with gastritis receiving gastric endoscopy and found *Proteobacteria*, *Bacteroidetes*, *Firmicutes*, and *Actinobacteria* together accounted for 99.82% of the gastric microbiota [38], the interaction network between microbiota significantly changes in *H. pylori* positive individuals with an enrichment in fifty-five gastric microbial pathways. In *H. pylori* negative individuals, co-abundance networks between species were negatively associated with *Stenotrophomonas maltophilia*, while in *H. pylori* positive individuals this relationship turned positive. Miftahussurur *et al.* identified the families *Streptococcaceae* (25.9%), *Prevotellaceae* (13.5%), and *Veillonellaceae* (10.8%) predominated in the *H. pylori*-negative group in the gastric mucosa of Indonesian individuals with gastritis or intestinal metaplasia [39]. Other genera detected as part of the *H. pylori* negative individuals with gastritis gastric microbiota were *Rothia*, *Fusobacterium* [25], *Gemella* [43]*, Haemophilus* and *Porphyromonas* [37], [38]. All negative associations involved *H. pylori*, indicating restructuring of the gastric microbiome in *H. pylori* positive cases. One study showed that the bacterial load in patients with gastritis is higher than in controls without an elevated pH [37], suggesting that a higher pH may be associated with bacterial overgrowth in the stomach [31], [32]. A significantly higher abundance of the genus *Streptococcus* was detected in a less acid milieu, which may be linked to the development of peptic ulcer disease [44]. The potential role of non-*H. pylori* species in the pathogenic process of gastritis still requires further investigation in larger cohorts.

Two studies included 56 and 5 *H. pylori* negative individuals (healthy individuals without *H. pylori* infection) [45], [46], Yuan *et al.* investigated both gastric mucosa and gastric fluid samples in Chinese individuals of healthy and chronic gastritis patients combined. *Streptococcus*, *Helicobacter*, *Neisseria*, *Alloprevotella*, *Prevotella*, and *Actinomyces* were the most dominant taxa in gastric mucosal samples in *H. pylori* negative individuals [45]. Monstein *et al.* have analyzed the gastric microbiome by using temporal temperature gradient gel electrophoresis (TTGE) and a small-scale 16 S rDNA sequence showed that in addition to *H. pylori*, other microbes such as *Enterococcus*, *Pseudomonas*, *Streptococcus*, *Staphylococcus*, and *Stomatococcus* were present in the gastric mucosa samples [46]. *Helicobacter* sequences were detected in all *H. pylori*-negative gastric biopsies suggesting asymptomatic colonization, which might be another feature of this younger age cohort.

Two studies from Li *et al.* and Delgado *et al.* focused on *H. pylori* negative gastritis and found that the abundance of *Streptococcus* was significantly higher in this group [5], [37]. In addition, Li *et al.* cultivated bacteria from the biopsies and washed biopsies with phosphate buffered saline (PBS) to verify the bacteria of *Streptococcus* phylotypes are indeed resident instead of passersby in the stomach [5], [37].

In conclusion, the most abundant gastric microbiota detected by 16 S rRNA sequence analysis in the *H. pylori* positive gastritis individuals belongs to the phyla *Firmicutes*, *Actinobacteria*, *Bacteroidetes*, *Fusobacteria*, and *Proteobacteria* and the most prevalent genera in *H. pylori* negative gastritis are *Prevotella*, *Streptococcus*, *Veillonella*, *Rothia*, and *Haemophilus* (Table 1, Fig. 2). Compared to *H. pylori* positive individuals, negative individuals had increased levels of *Streptococcus* [5], [37], [39], [43], [45], *Prevotella* [37], [39], [43], [45], *Neisseria* [37], [45], and *Actinomyces* [43], [45], and 2 out of 3 studies indicate that the microbiota relative abundance can be restored after *H. pylori* eradication.

### 3.3 The influence of eradication therapy and PPI use on the gastric microbiome in relation to GC

#### 3.3.1 *H. pylori* eradication therapy and PPI treatment

A standard bismuth-containing quadruple (PPI, bismuth, tetracycline, and metronidazole) and clarithromycin triple (PPI, clarithromycin, and amoxicillin) therapy has been recommended as a first-line treatment for *H. pylori* infection by the Maastricht VI/Florence Consensus Report [47]. A recent meta-analysis including nine studies with 546 participants describes the impact of first-line *H. pylori* eradication treatment modalities on the microbiome of the stomach [48]. Shortly, quadruple therapy temporarily alters α-diversity after short-term (1–2 month) and long term (>6 months) follow-up, which was similar in the triple therapy group. The microbiota composition changed after eradication resulting in a significant decrease in the relative abundance of *H. pylori* and an increase in *Firmicutes*, *Bacteroides* and *Actinobacteria* for quadruple therapy [40], [41], [45], [49] and triple therapy [50], [51], [52], [53]. No significant difference between the quadruple and triple modalities were observed. Importantly, while after short-term follow-up a higher diversity compared to non-*H. pylori* individuals was still observed in individuals treated with quadruple therapy [45], α-diversity was at similar levels in the post-eradication groups compared to non-infected individuals after long-term follow-up [48]. These studies show that *H. pylori* eradication can successfully reconstitute the microbial composition in the stomach to levels of *H. pylori* negative individuals. In addition, the use of probiotics may help in restoration of the gastric microbiome after eradication therapy [45].

Although PPIs are included in eradication treatment of *H. pylori,* resulting in restoration of the gastric microbiome, PPIs are also commonly used in the treatment of non-erosive reflux disease (NERD), gastroesophageal reflux disease (GERD) and as a stomach protector when patients use NSAIDS/ASCAL. Commonly prescribed PPIs include omeprazole, esomeprazole, pantoprazole, and lansoprazole, amongst others, that are indicated as first-line therapy in NSAID-related ulcers and GERD [54], [55]. PPIs reduce the acidity of the stomach from< pH 2 to >pH4. This change in pH is related to changes in the gastric microbiome as well as the fecal microbiome, potentially due to a higher passing and survival of microbiota from the oral cavity [56]. When long term PPIs are used, PPIs block the gastric acid secretion by irreversibly inhibiting the H^+^/K^+^ adenosine triphosphatase pump, resulting in increased gastrin secretion and elevated systemic gastrin levels. It is important to note that *H. pylori* may change its location after long-term PPI therapy. There is a transition from antral-dominant gastritis to corpus-dominant gastritis, which inhibits gastric acid secretion and stimulates increased gastrin secretion by G cells. Gastrin increases gastric motility but also enhances mucosal growth via stimulation of gastric mucosal endocrine cells (ECLs), which may be related to the development of ECL cell hyperplasia and neuroendocrine tumors (NETs) [57], [58].

#### 3.3.2 GC risk after long-term PPI use

Multiple studies suggest that long-term use of PPIs (≥12 months) is associated with a higher risk of GC, and long-term prescription of PPIs should be avoided [59], [60], [61], [62]. Seo *et al.* used a national database, which included medical information on more than one million individuals [62]. They assessed the association between the use of PPIs and GC. A 2.37-fold increase in the incidence of GC when using PPIs for longer than 30 days was found. Next to that, they found that PPI use over 180 days was linked with a 2.22-fold increase in the incidence of GC after *H. pylori* eradication. Likewise. Segna *et al.* performed a systematic literature review with meta-analysis and concluded that PPI use was associated with a two-fold increase in GC incidence [63]. The most plausible hypothesis for the association between long-term PPI intake and the development of GC is mediated via hypergastrinemia due to the reduced secretion of gastric acid [64]. Guo *et al.* reported in their meta-analysis (including 24 studies, n = 8066349) there was a significant positive correlation between the use of PPI and the risk of non-cardia GC and a trend between the duration dependent effect of PPI use and the risk of GC (<1 year RR = 1.56, 95% CI: 1.30–1.86; 1–3 years RR = 1.75, 95% CI: 1.28–2.37; >3 years RR = 2.32, 95% CI: 1.15–4.66) [65]. The patients that used PPI for more than 3 years had the highest risk of GC, which may be related to hypergastrinemia. Subgroup analysis correcting for potential confounders, most importantly *H. pylori* infection, showed a near 3-fold increase in GC risk. However, the exact mechanism how PPI use might directly or indirectly via microbiota or hypergastrinemia lead to GC is currently unknown. One suggestion is that use of PPIs or *H. pylori* eradication might create an environment for N-nitrosamine formation via non-*H. pylori* microbiota. However, the current results only showed a possible association between PPI use and GC, however, a direct causative link has not been reported so far [64].

#### 3.3.3 Changes in gastric microbiota due to PPIs

PPIs are frequently prescribed and long-term use of PPIs is a growing concern as it may disrupt one of the important barriers of the body against pathogens, with a potential increased risk of enteral infections [56]. PPI use could alter gut microbiota toward a dysbiotic state characterized by decreased diversity and increased pathogenic bacteria taxa and it is associated with inflammation, and accumulation of nitrogenous products [65].

PPIs can affect gastric microbiome diversity by directly targeting bacterial and fungal proton pumps or disrupting the normal gastric microenvironment by increasing gastric pH; changes in the gastric microbial composition occur principally in the number of bacteria that can increase by a factor of 200 due to the more favorable pH [31], [66], [67]. Compared to combining antibiotics therapy, there are fewer studies exploring the effects of PPI used alone on the gastric mucosal microbiome. Adamsson *et al.* and Mowat *et al.* found an increase in the diversity and abundance of gastric microbiota in individuals treated with PPI [68], [69]. Parsons *et al.* indicated that *Cyanobacteria,* and *Streptococcus* were significantly increased in the PPI-treated samples, whereas *Prevotella, Porphyromonas, Treponema*, *Leptotrichiaceae*, *Haemophilus*, and *Fusobacterium* were significantly decreased compared to normal stomachs [70]. Sterbini *et al.* indicated that *Streptococcus* was significantly increase in PPI-treated patients, and this increase seemed to occur independently of *H. pylori* [71]. Despite the use of gastric fluid samples, the increased abundance of *Streptococcus* was confirmed in patients taking PPIs by Rosen *et al.* [72]. In addition, this study found a significant correlation between PPI dose and abundance of *Streptococcus* [72]. Brush samples obtained from gastric mucosa of patients on continuous PPI treatment showed an increased abundance of oral strains of *Streptococcus*, *Neisseria* and *Haemophilus influenzae* upon culturing compared to individuals not on PPIs [73]. In contrast, Tsuda *et al.* showed that there was no significant difference between PPI users and non-PPI users in gastric juice samples, however, PPI users usually had a lower gut microbial diversity [74]. Importantly, the results from gastric juice might be different and less relevant when investigating GC development than those from brush samples and mucosal biopsies[75]. Some studies show only small significant differences in the microbiome composition between non-PPI users and patients receiving PPIs [71], [76], [77]. Small sample sizes in these studies may have led to variation and inability to observe differences between groups.

In conclusion, the α-diversity in PPI users was significantly increased in individuals treated with PPIs and the genus *Streptococcus* was most consistently found to be increased in abundance in PPI users (Fig. 2). Whether these changes and increased bacterial abundance in the stomach is related to the increased risk for GC in PPI-users need to be investigated, preferably in longitudinal studies with larger sample sizes.

### 3.4 The influence of obesity on GC and the gastric microbiome

#### 3.4.1 GC risk in obese individuals

Obesity (BMI>30) affects hormonal levels, including insulin and estradiol, which are thought to contribute to the onset of several cancers [78]. A study performed by EPIC (European Prospective Investigation into Cancer and Nutrition) incorporated 476.160 participants who investigated the relation between anthropometric and reproductive factors to GC [79]. The cancers were classified as either gastric cardia cancer or gastric non-cardia cancer. A positive association between BMI and gastric cardia cancer was found, but not for non-cardia GC. A meta-analysis performed by Turati *et al.* identified 22 studies and also concluded that obesity is related to gastric cardia adenocarcinomas [80]. In addition, Lauby-Secretan *et al.* confirmed this positive link between BMI and the development of gastric cardia cancer [81], however, there was inadequate information for non-cardia cancer to perform the calculations and whether to confirm or deny the results of Sanikini *et al.* and Lauby-Secretan *et al.* The exact mechanism how obesity promotes GC is not clear yet. Multiple mechanisms may play a role; the increased fat deposition may affect DNA repair as well as cell proliferation via hyperinsulinemia, abnormal high Insulin-like Growth Factor (IGF)-levels, or an imbalance in adipokines. It is known that insulin can stimulate gastric adenocarcinoma cell PI3-kinase/Akt signal transduction, proliferation, and survival [82]. In a nested case–control study conducted in Japan a positive association between circulating insulin levels and GC development was reported supporting this hypothesis [83], [84]. Also, obesity-induced inflammation creating a chronic subclinical pro-inflammatory state with increased pro-inflammatory cytokines leading to oxidative stress may play a role. In addition, estrogen levels have been implicated in GC development. First the incidence of GC increases in post-menopausal women [15], and second, the use of menopausal hormone therapy including estrogen or estrogen with progesterone reduces the risk for GC [85], suggesting that hormonal balance might be an important factor in GC-risk. Together, this suggests that obesity-related GC is most-likely a multifactorial event [78].

#### 3.4.2 Gastric microbiome in obese individuals

The gut microbiome has been identified as an important factor associated with obesity. In non-obese individuals, about 90% of the bacterial taxa belong to the phyla *Firmicutes* and *Bacteroidetes*, followed by *Actinobacteria*, *Proteobacteria*, and *Verrucomicrobia*. Alterations in gastric microbiota in patients with obesity is not fully understood yet. The only study published so far by Carolina Gutiérrez-Repiso *et al.* included 41 morbid obese individuals (BMI > 40 kg/m2 or BMI >35 kg/m2 with comorbidities) who underwent sleeve gastrectomy. The gastric mucosal samples obtained during the operation were analyzed by high-throughput-sequencing and showed *Streptococcaceae*, *Bacteroidaceae*, *Prevotellaceae*, *Carnobacteriaceae*, *Enterobacteriaceae*, *Micrococcaceae*, *Neisseriaceae*, and *Lachnospiraceae* were the predominant bacteria at family level. At the genus level, *Streptococcus* was the genus most represented, followed by *Bacteroides, Prevotella, Alkalibacterium*, *Rothia*, *Neisseria*, *Shewanella*, *Pseudomonas*, and *Actinomyces.* Finally, at the species level, *Bacteroides* species were the most predominant species, followed by *Rothia mucilaginosa*, *Alkalibacterium olivapovliticus*, *Faecalibacterium prausnitzii*, *Prevotella copri*, and *Actinomyces odontolyticus* [77]*.* However, the effect of obesity on the human gastric mucosa microbiome have not yet been studied by others and no comparative studies between obese and healthy individuals have been performed yet.

#### 3.4.3 Gastric microbiome in animal models

Although there are few studies of human gastric mucosa in obese individuals, several animal studies were conducted. He *et al.* showed that the gastric microbiome changes in 24 weeks high fed diet (HFD) fed mice with especially a decrease in *Akkermansia muciniphila*, a beneficial bacterium with probiotic properties. Some interventional studies have even shown that daily administration with *A. muciniphila* can counteract the negative effects on metabolism of a HFD diet in mice [86]. Besides the decline in *A. muciniphila*, the 24 weeks-HFD mice had higher abundance of *Lachnospiraceae*, *Rikenellaceae*, and *Desulfovibrio*, which have been reported to be positively correlated with obesity [87]. A mice study by Arita *et al.* tested several HFDs, including those based on lard, coconut, linseed oil, corn oil and cocoa butter. All HFDs increased microbial numbers in the stomach and led to a higher abundance of *Lactobacillus*, although this varied by HFD-type. *Bifidobacteriales* was less abundant in the gastric microbiota and this reduction sustained at 12 and 24 weeks HFD [88]. A low abundance of *Bifidobacterium* has been associated with diet induced obesity in another study. Supplementing *Bifidobacterium* to obese mice resulted in a significant reduction of body and organ weights [89]. Interestingly, Arita *et al*. showed that intestinal metaplasia, a precursor of GC, shaped the microbial community, and its occurrence was independent of BMI and composition of the microbial community, but depended on HFD-induced gastric leptin signaling. These results suggest that microbiota changes may follow the early steps in carcinogenesis in this obesity model and that intestinal metaplasia may be driven by changes in leptin signaling [88], [90]. Whether the alterations in the microbial community within the stomach are linked to a high-fat diet, correlated with gastric leptin, or relate to human gastric carcinogenesis in obesity requires further exploration.

### 3.5 The gastric microbiome in GC

A chronic *H. pylori* infection or the use of drugs like PPIs can cause hypochlorhydria (pH between 4 and 7), which results in a more favorable environment for certain non-*H. pylori* bacteria [23], [91]. The colonization of other microbes in a less acidic environment may lead to dysbiosis, bacterial overgrowth and, via interaction with the gastric mucosa, could increase the risk of GC. Up to now, 18 human studies have analyzed the composition of the gastric microbiome in patients with GC (Table 2).

**Table 2:** GC microbiota detected by 16 s rRNA sequencing analysis.

Study reference	Individuals	Dominant microbiota in GC		Detection method/ Platform	Tissue type
Kim *et al.* (2022)[105]	GC(N = 45) CG(N = 49) IM(N = 43)	Genus level	Species levels	16 S rRNA sequencing /Illumina 16 S MiSeq platform	Biopsy
		*Lacticaseibacillus↑* *Haemophilus↓* *Campylobacter↓*	*L. casei↑* *Haemophilus parainfluenzae↓*		
Abate *et al.* (2022)[93]	GC(N = 520) Non-malignant tissue(N = 520)	Increase in the abundance of *Helicobacter*, *Lactobacillus*, *Streptococcus*, *Prevotella*, *Bacteroides* compared to non-malignant tissue		Next-generation sequencing/ IMPACT assay platform	Biopsy
He *et al.* (2022)[103]	SG(N = 61) GC(N = 64)	Increase in the abundance of *Lactobacillus*, *Prevotella*, *Fusobacterium*, *Veillonella*, *Neisseria*, *Sarcina*, *Alloprevotella*, *Gemella*, *Leptptrichia*, *Dolosigranulum*, *Selenomonas* and *Lachnoanaerobaculum* compared to SG		16 S rRNA sequencing/ Illumina Miseq instrument at BGI	Biopsy
Sun *et al.* (2022)[99]	CG(N = 65) IM(N = 27) GC(N = 13)	*Streptococcaceae*(9.21%−23.92%) and *Lactobacillaceae* (6.42%−8.61%)in GC lesions as a distinguishable bacterial taxon		16 S rRNA sequencing/ Quantitative Insights Into Microbial Ecology (QIIME) platform	Biopsy
Liu *et al.* (2021)[100]	CG(N = 176) GC(N = 657)	Increase in the abundance of *Gemella*, *Veillonella*, *Streptococcus*, *Actinobacillus*, and *Hemophilus* compared to CG		16 S rRNA sequencing/ NovaSeq PE250 platform.	Biopsy and datasets
Tseng *et al.* (2016)[67]	GC (N = 6)	Phylum level	Genus level	16 S rRNA sequencing/ Illumina MiSeq 2000 sequencer with MiSeq Reagent Kit v3 (Illumina)	Biopsy
		*Proteobacteria* *Actinobacteria* *Firmicutes* *Bacteroidetes*	*Ralstonia* *Helicobacter* *Lactobacillus* *Stennotrophomonas*		
Wang *et al.* (2016)[92]	CG (N = 212) Non-cardia GC (N = 103) Intestinal type (N = 87) Diffuse type (N = 16)	Phylum level	Genus level	16 S rRNA sequencing/ 454 GS-FLX system	Biopsy
		*Firmicutes, Bacteroidetes, Actinobacteria, Proteobacteria, Fusobacteria*	Increase in the abundance of *Lactobacillus*, *Escherichia Shigella*, *Nitrospirae*, *Burkholderia fungorum*, *Lachnospiraceae* in GC patients (*Nitrospirae* was not present in chronic gastritis)		
Dicksved *et al.* (2009)[17]	Noncardia GC (N = 10) Intestinal type (N = 5) Diffuse type (N = 5) Dyspepsia (N = 5)	Phylum level	Genus level	T-RFLP, 16 S rRNA sequencing/ ABI 3700 capillary sequencers (Applied Biosystems)	Biopsy
		*Firmicutes, Bacteroidetes, Actinobacteria, Proteobacteria, Fusobacteria* Low abundance of *H. pylori* in all samples	*Streptococcus, Lactobacillus, Veillonella, Prevotella*, *Neisseria*, and *Haemophilus* no significant difference from the control group.		
Eun *et al.* (2014)[19]	GC (N = 11) IM (N = 10) CG (N = 10)	Increase of *Lactobacillus* in GC compared to chronic gastritis group. Increase of *Streptococcus* and a decrease of *H. pylori* in GC compared to chronic gastritis and IM group.		16 S rRNA sequencing/ 454 GS FLX Titanium sequencing with 454 Life Sciences Genome Sequencer FLX	Biopsy
Aviles-Jimenez *et al.* (2014)[18]	GC (N = 5) IM (N = 5) NAG (N = 5)	Increase in the abundance of *Lactobacillus* and *Lachnospiraceae* in GC compared to IM and NAG Decrease in the abundance of *Porphyromonas, Neisseria* in GC compared to IM and NAG		16 S rRNA sequencing	Biopsy
Jo *et al.* (2016)[116]	GC with *H. pylori* (-) (N = 19) Healthy with *H. pylori* (-)	Nitrate-reducing bacteria other than *H. pylori* was two times higher in cancer patients than in healthy individuals. No statistically significant result.		16 S rRNA sequencing	Biopsy
Ferreira *et al.* (2018)[101]	GC (N = 54) CG (N = 81)	Phylum level	Genus level	16 S rRNA sequencing	Biopsy
		*Proteobacteria, Firmicutes, Bacteroidetes, Actinabacteria, Fusobacteria*	Decrease in the abundance of *Helicobacter* in GC. Increase in the abundance of *Citrobacter*, *Clostridium*, *Lactobacillus*, *Achromobacter*, and *Rhodococcus* in GC.		
Coker *et al.* (2018)[94]	SG (N = 21) AG (N = 23) IM (N = 17) GC (N = 20)	Increase in the abundance of genera *PeptoStreptococcus*, *Dialister* and *Mogibacterium* in GC compared to SG, AG, and IM Increase in the abundance of Prepto*Streptococcus stomatis*, *Streptococcus angionasus*. *Parvinomonas micra*, *Slackia exigua* and *Dialister pneumosintes* in GC patients		16 s rRNA sequencing	Biopsy
Gunathilake *et al.* (2019)[108]	GC (N = 268) Healthy (N = 288)	Genus level	Species level	16 s rRNA sequencing	Biopsy
		Increase in the abundance of genera *Helicobacter*, *Propionibacterium*, and *Prevotella* in GC. Decrease in the abundance of genera *Lactococcus* in GC	Increase in the abundance of *H. pylori*, *Propionibacterium acnes*, and *Prevotella copri* in GC compared to healthy controls. Decrease in the abundance of *Lactococcus lactis* in GC compared to healthy controls		
Shao *et al.* (2019)[97]	GC (N = 36) Paired non-tumor sample(N = 36)	Increase in the abundance of *Prevotella, Streptococcus, Veillonella, Haemophilus, and Neisseria* in GC. Decrease in the abundance of the *Helicobacter* in GC compared to non-tumor tissue.		16 s rRNA sequencing	Biopsy from resection
Zhang *et al.* (2021)[98]	GC (N = 33) Healthy (N = 32)	Increase in the abundance of *Leptotrichia, Fusobacterium Prevotella, Porphyromonas, Capnocytophaga, Lactococcus, Streptococcus, Bacillus, Selenomonas, Peptoniphilus, Anaerococcus, Finegoldia, Methylobacterium, Proteus, Aggregatibacter, Haemophilus, Kingella, Neisseria, Cardiobacterium, DA101 (Verrucomicrobia phylum), Nitrospira, Candidatus Solibacter, Corynebacterium, Actinomyces, Bifidobacterium, Atopobium,* and *Thermus* Decrease in the abundance of *Megamonas.*		16 s rRNA sequence	Stool samples
Hsieh *et al.* (2018)[102]	GC (N = 11)	Increase in the abundance of phylum *Chloroflexi, Verrucomicrobia, Acidobacteria, Planctomycetes, Nitrospirae, Actinobacteria, TM7,* and *Fusobacteria* in GC compared to healthy	Increase in the abundance of genera *Clostridium*, *Fusobacterium*, *Lactobacillus* in GC compared to healthy	16 S rRNA sequencing	Biopsy
Sohn *et al.* (2017)[110]	GC with *H. pylori*(+) (N = 2) healthy with *H. pylori* (-) (N = 2)	Increase in the abundance of *S.pseudopneumoniae*, *S.parasanguinis* and *S.oralis* in *H. pylori* positive GC patients		16 S rRNA sequencing	Biopsy

NCGC- non-cardia GC, IM – intestinal metaplasia, IGC – intestinal GC, NAG – Non-atrophic gastritis, GCA – gastric cardia adenocarcinoma, GC – gastric cancer, CG – chronic gastritis, SG – superficial gastritis, AG –atrophic gastritis, CSG – Chronic superficial gastritis,

At the phylum level, the composition of the microbial community in GC is similar to chronic gastritis. Thus, regardless of mucosal state, the most prominent phyla are *Proteobacteria*, *Bacteroidetes*, *Fusobacteria*, *Actinobacteria*, and *Firmicutes* [17], [18], [25], [67], [92]. The most prominent genera in GC patients were *Streptococcus* [93], [94], [95], [96], [97], [98], [99], [100], *Lactobacillus* [18], [92], [95], [96], [99], [101], [102], [103], *Prevotella* [93], [95], [97], [98], [103], [104], *Neisseria* [18], [95], [97], [98], [103], *Haemophilus* [95], [97], [98], [100], [105], and *Veillonella* [95], [97], [103]. The diversity of gastric microbiota decreases with the progression from gastritis to intestinal metaplasia, and GC [16], [18], [67], [99], [105], [106], [107].

Recently, Shao *et al.* found that the relative abundance of *Helicobacter* decreases and *Prevotella* increases with more advanced tumor stage in gastric cardia adenocarcinomas (GCA) compared to non-tumor tissue. Also Dicksved *et al.* [17] found a relatively low abundance of *H. pylori* and a domination of the GC microbiome by different species of the genera *Streptococcus*, *Lactobacillus*, *Veillonella* and *Prevotella*. In contrast, Gunathilake *et al.* found *Helicobacter* to be the dominant taxa in GC patients, followed by *Propionibacterium*, and *Prevotella*, and a relatively high abundance of the species *Lactococcus lactis* was found in the control groups. *Propionibacterium lactis* was verified to be increased in GC patients and was shown to have an anti-proliferative activity in human colorectal cancer cells. The lowered relative abundance of *P. lactis* in the GC group suggests it may serve as a protective factor against the development of GC [97], [108]. In addition, the expression of p53 and p21 can be affected by *Lactococcus lactis* ssp. *lactis*, which can induce apoptosis and cell cycle arrest [109]. Eun *et al.* performed a separate analysis in the patients with *Helicobacter* dominance status and found that the composition of microbiota in this *Helicobacter*-dominant group was significantly different between GC patients and controls with a relative increase in *Streptococcus* family [19].

The largest GC population to date was investigated by Abate *et al.* including the IMPACT cohort with 234 GC and 40 tumor adjacent non-malignant samples and the TCGA cohort revealing 286 samples with microbial sequencing information. There were five bacterial taxa significantly enriched in both cohorts compared to non-malignant tissue, including *Bacteroides, Helicobacter, Lactobacillus, Prevotella* and *Streptococcus.* There were no significant differences in enrichment between intestinal and diffuse type GC. However, *Lactobacillus, Streptococcus, Prevotella, Fusobacterium, Selenomonas,* and *Porphyromonas,* were enriched in microsatellite instability-high GC [93].

Several studies found an increased abundance of lactic acid bacteria in GC patients. An increase in *Lactobacillus* was reported in 11 of 18 studies [18], [19], [67], [92], [93], [99], [100], [101], [102], [103], [105], and an increase in *Streptococcus* in 8 of 18 studies [19], [93], [94], [97], [98], [99], [100], [110]. Sun *et al.* investigated the patients with *H. pylori-*negative GC, and identified *Lactobacillus*, *Bifidobacterium* and *Streptococcus* as the most relevant taxa for GC [99]. Kim *et al.* found that *Lacticaseibacillus*, which belongs to the lactic acid bacteria (LAB), was significantly enriched in the GC group, while *Haemophilus* and *Campylobacter* were depleted and together were able to distinguish GC patients from controls. Interestingly, these discriminating bacterial taxa were significantly correlated with increased expression of mucosal IL1B mRNA expression. Previously, Korean researchers suggested that the presence of IL1B–31 T/IL1B–511 C polymorphism increased the production of IL1B by the gastric mucosa in *H. pylori* infection, and consequently, could promote the development of the intestinal type GC [105].

*Prevotella* was also enriched in gastric adenocarcinoma. The LPS-producing bacteria *Prevotella melaninogenica, Neisseria sicca,* and *Veillonella parvula* were significantly increased in bile reflux gastritis (BRG) and GC, with *Prevotella melaninogenica* having the highest relative abundance in both groups [111]. Bile reflux promotes gastric carcinogenesis via elevated levels of bile acids (BAs) and lipopolysaccharide (LPS) via these LPS-producing bacteria. Although these results were obtained from a mice model, it provides us a way to investigate potential causality in the onset of GC of microbial associations discovered in human gastric microbiome studies. For example, non-*H. pylori* gastric microbiota was isolated with culturing methods using selective media and subsequently cocultured with gastric epithelial cell lines. In these cell models, LPS from *Neisseria subflava* interacted with toll-like receptor 4 (TLR4), resulting in potent stimulation of IL-8 expression, and may thus contribute to gastric inflammation. *N. subflava* colonized in the gastric mucosa may perpetuate gastric inflammation and accelerate neoplastic progression in the hypochlorhydric stomach [112]. These microorganisms not only create a unique tumor micro-environment by altering the microbial niches in the mucosa, but they might also contribute to the onset of GC and accelerate its progression through inflammatory mediators, immune cells, or oncogenes.

An animal study which compared INS-GAS mice mono-infected with *H. pylori* to INS-GAS mice infected with *H. pylori* and other complex gastric microbiota resulted in a worse outcome in the combined model in the development of gastric carcinogenesis [113]. The non-*H. pylori* bacteria played a positive role in the development of GC by synergizing with *H. pylori*. Bacteria-host interactions and *H. pylori*-other bacteria microbial symbioses (when the mucosa colonized with *H. pylori*, with subsequent colonization of different, non-indigenous microbiota) are important causes of the development and progression of GC in this model. Based on these results we cannot directly conclude whether GC-enriched microbiota are a bystander or drivers of gastric carcinogenesis. However, we learned that the gastric mucosal microbiota changed significantly in the presence or absence of *H. pylori*, and this may contribute to the initiation of gastric carcinogenesis. Even in precancerous stages, because *H. pylori* and non-*H. pylori* bacteria are an integral part of the gastric micro-environment in gastric carcinogenesis. Exemplified by a recent study investigating the progression of precancerous lesions with network analysis, where they showed that microbial diversity decreases across precancerous stages from atrophic gastritis, intestinal metaplasia and intraepithelial neoplasia and that the gastric mucosal genera *Gemella, Veillonella, Streptococcus, Actinobacillus,* and *Hemophilus* had the higher degree of centrality and strong co-occurrence interaction in gastric biopsies across these precancerous stages in *H. pylori* negative individuals [100].

In conclusion, GC patients have an altered microbial composition at the genus level, which may be different between patients with high and low *Helicobacter* presence. Especially the genera *Streptococcus* and *Lactobacillus* were found to be more prominent in GC patients in multiple studies. Further research with larger sample sizes is necessary to get more representative results of the GC population and assess its clinical relevance. Furthermore, the changes in microbiome composition in precancerous versus cancerous stages and subtypes of GC needs to be further investigated. Bacterial genera that showed a trend toward statistical enrichment in MSI-High GC subtype, such as *Fusobacterium*, *Bacteroides*, *Prevotella* and *Streptococcuss*, were also found to be enriched in MSI-High type colorectal cancer [93]. These bacteria play an important role in the development of MSI-high tumors, or a unique immune environment exists in MSI-high tumors that leads to a colonization by these microbes [114]. These changes preceding GC are necessary to further investigate and understand their relevance in GC development.

### 3.6 Potential mechanisms of lactic acid bacteria in GC development

**Fig. 3:**
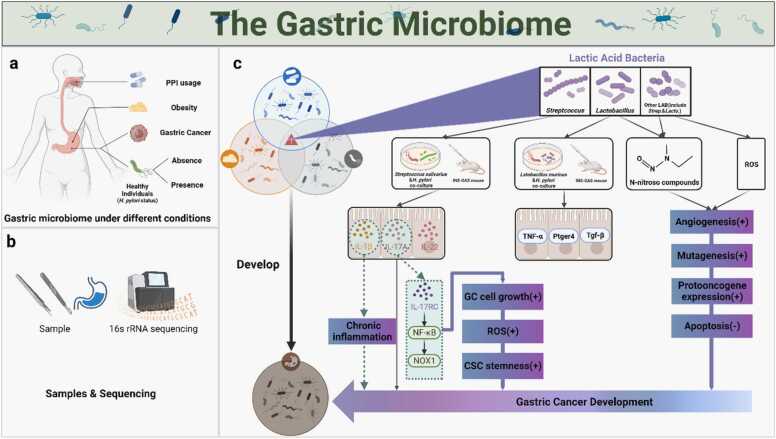
a. We investigated the gastric microbial composition of healthy individuals, PPI users, obese population, gastritis in presence/absence of *H. pylori*, and GC. b. Samples were obtained from gastric mucosal tissue, but we also discuss the impact of different tissue sources, such as gastric fluid or resections. Most studies used 16 s rRNA sequencing for the analysis of gastric microbiota. c. The microbiome composition varies between conditions, and all have the potential to influence the development of GC, with as commonality the increase in lactic acid bacteria, found in PPI use, obese individuals and non-*H. pylori* microbiome. The potential mechanisms of LAB may contribute to the development of GC.

The overview of the current studies reveals differences in microbiome composition and diversity in PPI users, obese individuals, *H. pylori* positive and negative healthy controls, and GC patients. Especially lactic acid bacteria (LAB), including *Lactobacillus* and *Streptococcus*, are increased in GC patients in multiple studies. Interestingly, the increase in *Streptococcus* is also observed in patients using PPIs and *Streptococcus* was the predominant bacterial genus in obese gastric microbiome in the only study performed so far. Therefore, there is a great need to explore the potential mechanisms of this group of bacteria (Fig. 3c).

The mechanism by which LAB promotes gastric carcinogenesis has not been extensively studied. *Lactobacillus*, a gram positive aerotolerant anaerobe, is often utilized as probiotics and assumed to be beneficial to the host, can produce lactate that can serve as an energy source for cancer cells, but also cancer cells can export lactate, which can acidify the tumor micro-environment and induce a local inflammatory response that attract immune cells, drives tumor cell growth and metastasis [115]. LAB can contribute to the formation of N-nitroso compounds [93], [116], and ROS, which promote mutagenesis, angiogenesis, protooncogene expression and inhibit apoptosis [117]. One study specifically focusing on nitrate reducing bacteria found an increase in GC compared to controls, however, this was insignificant and only considered 16 patients [87].

In previous studies, Lertpiriyapong *et al.* reported that colonization of male INS-GAS mice (INS-GAS transgenic mice overexpress pancreatic gastrin) with restricted microbiota consisting of *Lactobacillus murinus* ASF361, *Clostridium* ASF356, and *Bacteroides* ASF519, was sufficient to promote gastrointestinal intraepithelial neoplasia, which associated with robust expression of gastric inflammatory and cancer-associated genes, including TNF-α, Ptger4 and Tgf-β. The study suggested individuals presenting with gastric atrophy may be more susceptible to the detrimental effects of colonization by opportunistic microbiota [118].

In a recent study, *Streptococcus salivarius* and *H. pylori* were co-cultured and subsequently inoculated into mice. Compared to *H. pylori* mono-infection, the co-infection resulted in a robust pathological outcome. At the same time, the expression levels of IL-1β, IL-17A, and IL-22 were found to be significantly elevated in mice co-infected with *H. pylori* and *S. salivarius*, and Ki67 staining detected a significant increase in the number of proliferating epithelial cells in the stomach [119]. Kim *et al.* observed mucosal IL-1β mRNA overexpression had a positive correlation between the relative abundance of *Lacticaseibacillus* and it contributes to the development of GC by inducing chronic inflammation in *H. pylori*-negative group. Another study demonstrated that IL-17A can inhibit apoptosis and promote ROS production, sphere formation ability of cancer stem cells, and expression of stemness-related genes in gastric cancer AGS cells through the regulation of the IL-17RC/NF-ᴋB/NOX1 pathway [120].

*H. pylori* chronic infection plays an important role in chronic gastritis and atrophic gastritis and increases the risk for GC, however, its colonization in intestinal metaplasia and GC is scarce [121], highlighting a temporal relation of *H. pylori* with GC development increasing the recognition of the role of non-*H. pylori* factors in GC progression. Once LAB colonizes in GC, it can potentially promote GC via 4 mechanisms: 1) via impact the release of interleukin-1β/17 A/22, 2) via IL-17RC/NF-ᴋB/NOX1 pathway, 3) via gastric inflammatory and cancer-associated genes TNF-α/Ptger4/Tgf-β, 4) via N-nitroso compounds and 5) ROS (Fig. 3c). Whether these mechanisms play a role in human GC development remains to be further investigated.

### 3.7 Limitations

Several studies have shown differences in the composition of the gastric microbiome between patients with GC, gastritis and healthy individuals, and PPI users with their paired controls. The composition and diversity of the gastric microbiome has been described in detail and in recent years the sizes of the cohorts are increasing with reduced sequencing costs. However, different samples sources (e.g.: feces, gastric juice/ gastric mucosa or swabs) will result in significant different composition and diversity of bacteria. At the same time, differences in patient population, ethnicity, socioeconomic status, age, lifestyle, and genetic variation also play an important role in the composition and changes of the gastric microbiome throughout life.

Most of the studies reviewed in this study belong to cross-sectional studies, therefore, should be interpreted with caution particularly with regards to causation relationships. To assess potential causal relationships bacterial colonization in the gastrointestinal tract and its fluctuations in relationship to precancerous gastric lesions and GC development would have to be studied longitudinally in different populations across the world, including eating habits, living environment, medication and other variables influencing GC risk. These studies could reflect the gradual development of microbial dysbiosis before GC develops. Current developments in collection of longitudinal cohorts to study health effects could help shed light on these more complex health questions.

Technological differences are also an important factor in the inconsistent results of microbiome studies[122]. Although 16 s rRNA sequencing has given us a comprehensive map of the microbiome, variations in sample collection, contamination control, sample processing, DNA isolation, sequencing methodology and bioinformatic analysis can all impact the outcome of microbiome studies and influence the comparability between studies. Furthermore, it remains uncertain if the detected 16 s rRNA genes originate from dead or living microbiota, and whether they are living microorganisms in the stomach or have been collected in the sampling process in the endoscopy procedure. Lastly the abundance of the detected microbiota is uncertain as 16 s rRNA studies amplify the genes of interest. Especially low biomass samples such as gastric mucosa are highly influenced. 16 s rRNA analysis is non-specific as it amplifies the conserved microbial regions and sensitive to contamination with foreign microbial DNA. Sources of contamination include laboratory environment, DNA extraction reagents, and PCR reagents. It remains challenging to avoid sources of contamination. Studies investigating in situ microbiota detection in gastric biopsies might shed light on the actual localization and abundance of microbiota in the stomach in different clinical conditions.

## 4 Conclusions and future perspectives

Overall, most studies detected a comparable abundance of the most prominent phyla in the gastric microbiota. Differences in individual composition at the bacterial genus level may be due to geographical differences, technological differences, and the distribution of *H. pylori* inflammation worldwide. Nowadays, analysis of other gastric microbiota than *H. pylori* is possible through the innovation of 16 S rRNA sequence analysis.

So far, *H. pylori* remains the strongest risk factor for GC development. Besides *H. pylori*, GC patients may have a shift in the non-*H. pylori* gastric microbiome before the premalignant and malignant transformation of gastric mucosa occurs. Research has shown that despite the eradication of the main risk factor *H. pylori*, GC could not be prevented, which suggests a contribution of non-*H. pylori* factors to the development of GC. Eradication of *H. pylori* leads to restoration of the gastric microbiome, potentially faster when using probiotics, the GC microbiome differs from controls and between patients with and without *H. pylori* dominance.

PPIs are a common ingredient of *H. pylori* eradication treatment regimens and are frequently used to treat GERD or as stomach protector. An increase in pH induced by PPI use can lead to changes in the composition of the microbiome favoring towards a dysbiotic state characterized by decreased diversity and increased (opportunistic) pathogenic bacteria, especially increasing *Streptococcus spp*., and is associated with inflammation, and accumulation of nitrogenous products [65]. Because of the widespread use of PPIs, it is essential to further understand the impact of long-term usage for GC-risk and understanding the underlying mechanisms, if there are any.

Next to that, obese individuals have an increased risk for GC, potentially via a multitude of factors including low grade inflammation, leptin increase and influence on hormonal balance. This corroborates the findings in post-menopausal women treated with hormonal therapy reducing the risk for GC, especially through estrogen supplementation. However, how these factors influence the gastric microbiome of obese patients is not studied in detail so far.

Reviewing the literature, we found some studies showing that specific microbes had a strong relationship with GC, for example LAB, including *Lactobacillus* and *Streptococcus,* were increased in GC patients in multiple studies. These LAB may play an important role in the development of GC, because they are able to convert nitrogen to potentially carcinogenic N-nitroso compounds, promote ROS, regulate specific molecules to promote tumor development, or contribute to chronic inflammation as shown in mice models, which could explain an increased risk for GC.

There are still questions that remain unanswered. Studies that have analyzed the composition and the changes of the microbiome in GC patients are scarce, cross-sectional, and partially controversial. A powerful method of combining information from these different datasets across cohorts is the use of meta-analysis and statistical methods as recently performed in the study by Li *et al.*, showing an increased diversity after *H. pylori* eradication across cohorts as well as a consistent shift in mucosal microbiome in GC [123]. To understand a potential contribution of gastric microbiota beyond *H. pylori* in GC development, longitudinal studies including precancerous lesions, different regions in the stomach, and both intestinal and diffuse type GC should be performed. One of the current shortcomings is actual visual evidence of a gastric microbiome in mucosal samples. In our experience the bacterial abundance in gastric mucosal samples is low and this crucial information is relevant for our understanding of the impact on GC development. Importantly, the potential mechanisms of microbiota and influence of medication could be studied in gastric organoid models for diffuse and intestinal type GC [124]. The specific role of non-*H. pylori* microbiota in the pathogenesis of the development of different types of GC remains to be determined soon to get new insights in prevention strategies, management, and treatment of GC.

## Funding

This study was supported by 10.13039/501100004543China Scholarship Council (CSC) to Chengliang Zhou (Funding number: 202206180009).

## CRediT authorship contribution statement

**Chengliang Zhou** (First Author): Methodology;Investigation; Writing - Original Draft; **Tanya M. Bisseling** (Author 2): Data Curation; Writing - Review & Editing; **Rachel S. van der Post** (Author 3): Visualization; Investigation; Supervision；Writing - Review & Editing; **Annemarie Boleij** (Corresponding Author): Conceptualization; Resources; Investigation; Supervision; Writing - Review & Editing.

## Declaration of Competing Interest

The authors declare the following financial interests/personal relationships which may be considered as potential competing interests: Cheng-Liang Zhou reports financial support was provided by CSC.
