# Supplementary material for: The influence of Helicobacter pylori, proton pump inhibitor, and obesity on the gastric microbiome in relation to gastric cancer development
Source: Comput Struct Biotechnol J. 2023 Nov 30;23:186–98. doi: 10.1016/j.csbj.2023.11.053 (PMC10704269; doi:10.1016/j.csbj.2023.11.053)
Supplement: Supplementary file 1 — Supplementary material [file mmc1.docx]

# Supplemental figure

PRISMA flow diagram for selected literature for each condition
